# A Giant Epidermoid Cyst in the Floor of Mouth Mimicking Ranula

**DOI:** 10.7759/cureus.43741

**Published:** 2023-08-19

**Authors:** Vivek Dokania

**Affiliations:** 1 Department of Otolaryngology - Head and Neck Surgery, Asian Super Specialty Hospital, Dhanbad, IND

**Keywords:** ranula, dermoid cyst, head and neck, floor of mouth, epidermoid cyst

## Abstract

Dermoid cysts are benign ectodermal-derived epithelial cysts rarely found on the floor of the neck. They may be congenital or acquired. They comprise three histological variants according to their contents and include dermoid, epidermoid, and teratoma. Epidermoid cysts are lined by epithelium but do not contain skin appendages like hair follicles and sebaceous glands, as seen in dermoid cysts. Teratoma on the other hand contain mesodermal element. They reveal either a supra-myelohyiod or infra-myelohyiod floor-of-mouth location and can be clinically confused with various close differentials including infections, tumours, mucous extravasation phenomena, and embryonic abnormalities. A 28-year-old female presented with a complaint of painless large swelling beneath the chin. Computed tomography (CT) scan with contrast revealed a right para-median thick-walled cystic lesion located in the sublingual space. A plunging ranula was suspected on radiological assessment. Aspiration cytology revealed keratin-containing fluid and pointed towards a tentative diagnosis of dermoid/epidermoid cystic lesion. The mass lesion was explored via a transcutaneous neck approach. The final histopathological evaluation of the excised cystic lesion eventually confirmed a diagnosis of epidermoid cyst. Consider epidermoid cyst as a possible differential for any floor-of-mouth swelling. They can be clinically and radiologically confused with close differential including ranula, dentoalveolar cyst and lipoma. Aspiration cytology examination is sometimes helpful in equivocal cases. Cyst excision with histological examination allows for a confirmatory diagnosis and is possibly the only means of distinguishing between specific histological variants of dermoids.

## Introduction

Epidermoid cysts are epidermal inclusion cysts that can arise from developmental anomalies or an acquired traumatic process. Since they are categorized under dermoid cysts, no data is available about their incidence alone. Together, epidermoid and dermoid cysts comprise between 1.6 and 6.9 percent of all head and neck cysts [1]. They mostly occur in the third or fourth decade of life and show a slightly higher male predilection [2]. Mostly epidermoid cysts followed by dermoid cysts were reported to be found in the head and neck region; teratoid cysts are much rarer [3]. The various differentials of epidermoid cysts in the floor of the mouth include dermoid cysts, teratoid cysts, ranula, thyroglossal duct cysts, lipomas, lymphoepithelial cysts, various benign and malignant salivary gland tumours. Epidermoid cysts can often be confused with various close differentials, especially in cases with equivocal clinical and radiological presentation [4].

## Case presentation

**Figure 1:**
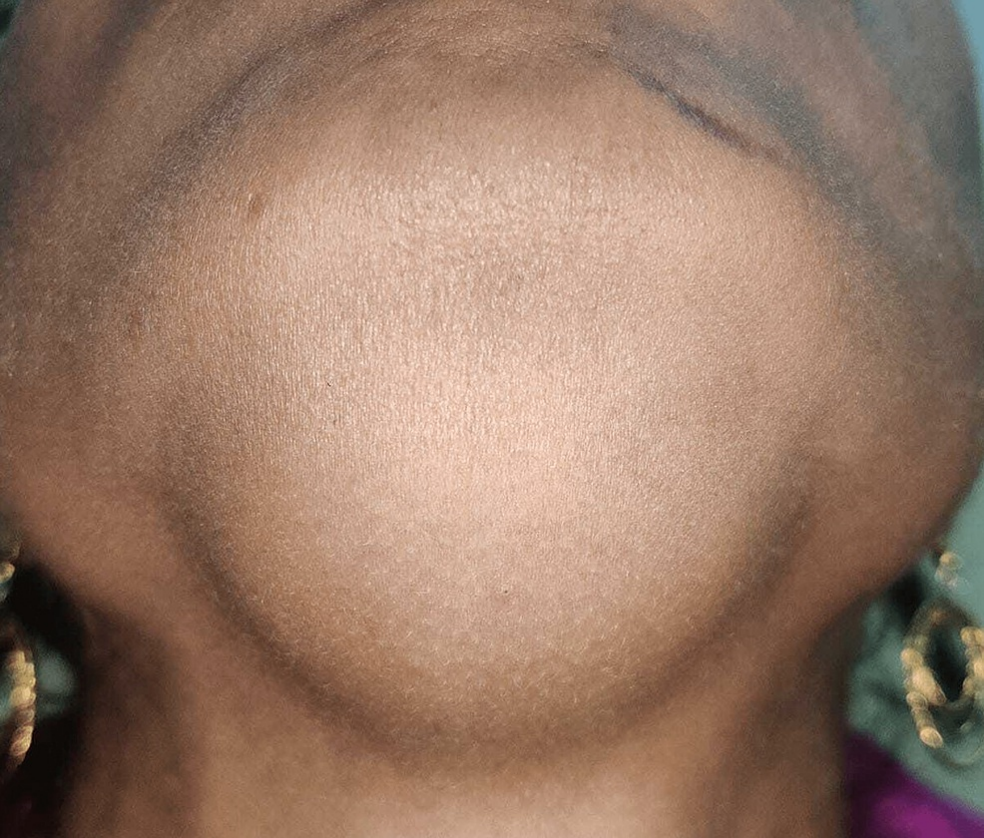
Clinical image showing a smooth swelling just beneath the chin

A 28-year-old female presented to our clinic with a complaint of a painless bulge beneath her chin of two years duration. An ENT examination revealed a soft, non-tender swelling in the submental region measuring approximately 4 x 3 cm with a smooth surface (Figure 1). Intra-oral examination showed a small bulge over the right paramedial floor of the mouth. The swelling did not transilluminate. The lesion did not move with tongue protrusion or with deglutition. No lymphadenopathies or other masses were noted on head and neck examination. No genetic or syndromic abnormalities were reported from her family.

**Figure 2:**
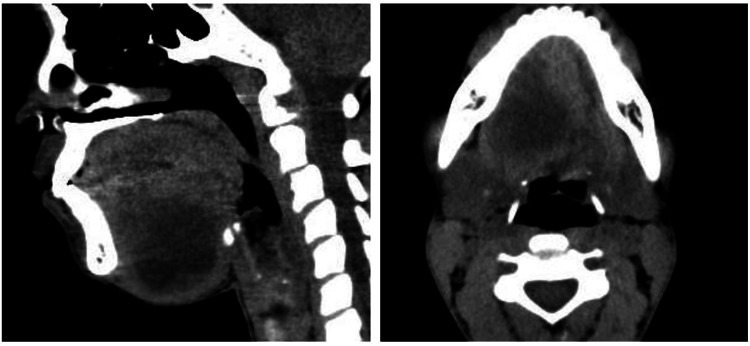
Computed tomography images of the neck Computed tomography images in sagittal (left-hand side image) and axial view (right-hand side image) show a homogeneous, well-defined lesion in the sublingual floor of the mouth region.

Computed tomography (CT) scans revealed a right para-medial thick-walled lesion in the sub-lingual space. The lesion was present just beneath the genioglossal muscle and abutted the anterior surface of the right submandibular gland. The lesion was well-defined, homogeneous, and measured 39 x 39 x 38 mm (Figure 2). A diagnosis of plunging ranula was offered on a CT scan. With a provisional diagnosis of plunging ranula, aspiration puncture was done. This showed a keratin-containing fluid, which raised the possibility of a new diagnosis of epidermoid or dermoid cyst.

**Figure 3:**
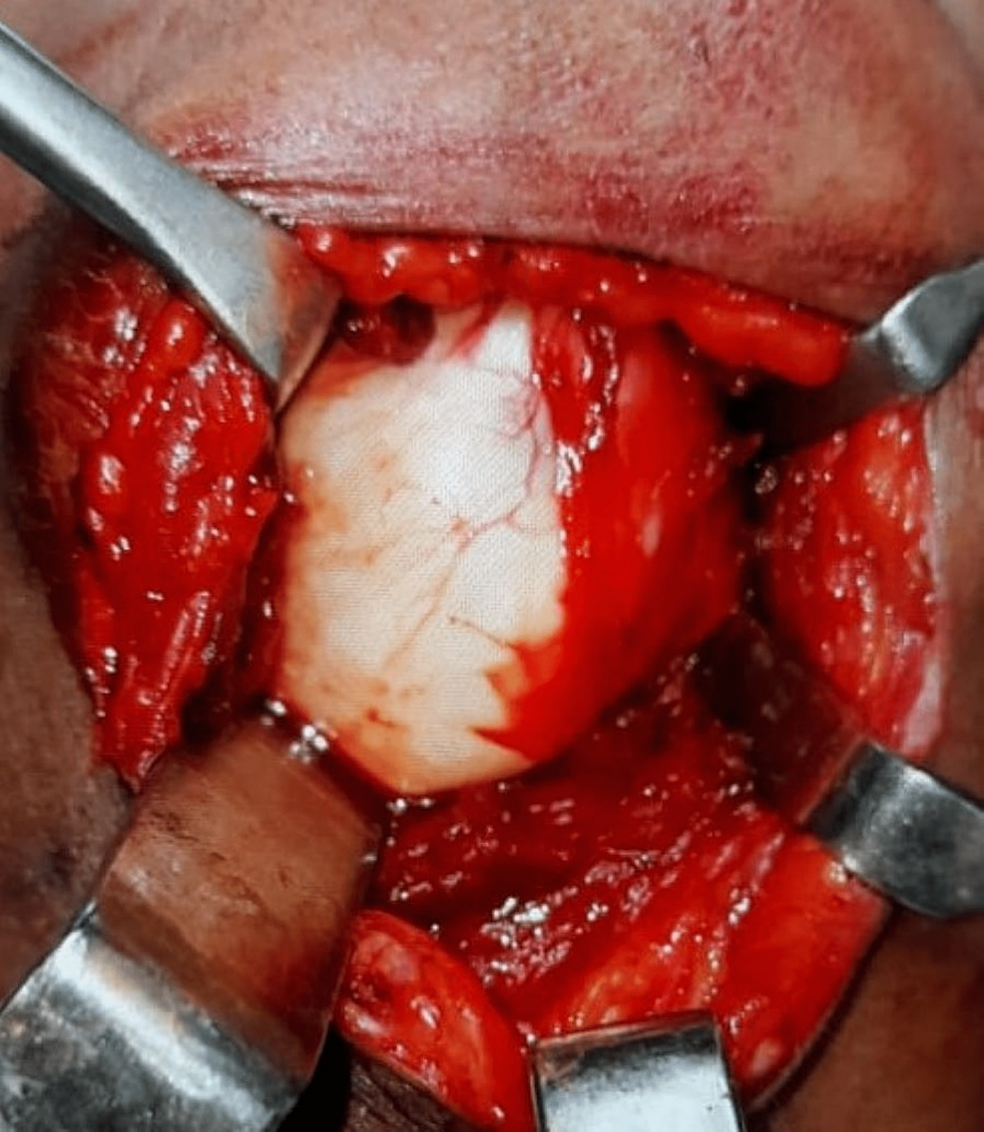
Intra-operative image of the cyst excised via a transcervical approach

The lesion was approached through the transcervical crescentic-shaped incision. The entire cystic lesion was delivered in toto after retracting the digastric muscle and separating from the underlying mylohyoid and geniohyoid muscle (Figure 3). The interior of the cyst did not contain any hair follicle or mesenchymal tissue but rather was filled with keratinous material.

**Figure 4:**
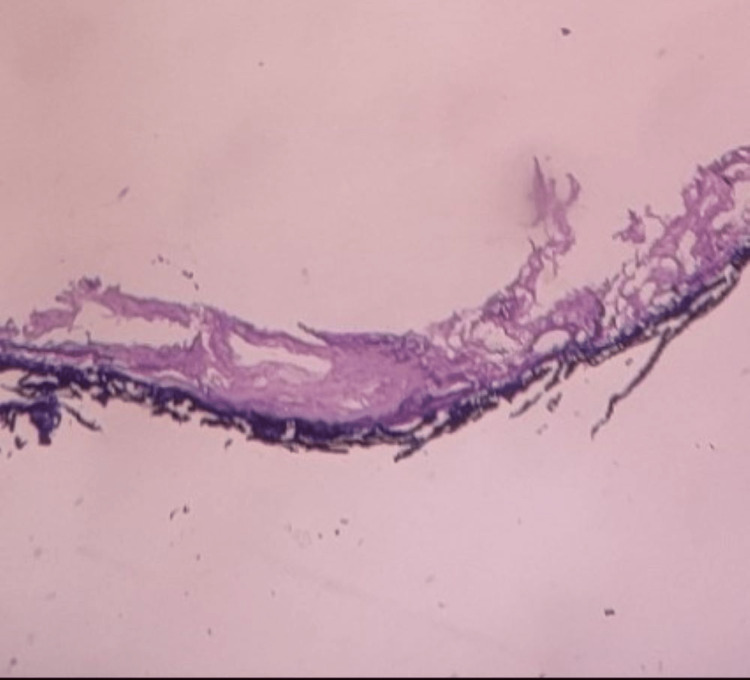
Histological study image The image shows an epithelial-lined cyst containing keratinous debris.

Histopathological evaluation of the excised lesion revealed a fibro-vascular cystic wall lined with squamous epithelium (Figure 4). No adnexal structure or thyroid tissues were found in the cyst. This confirmed a diagnosis of an epidermoid cyst on the floor of the mouth. No signs of recurrence were noted until the 18-month postoperative follow-up.

## Discussion

Various theories have been attributed to the development of epidermoid/dermoid cysts. Ectodermal entrapment during early foetal life has been most widely accepted, however, some consider these cysts to develop from pluripotent cells which get trapped during early embryonic life [5-7]. Acquired processes including traumatic or surgical implantation of epithelial tissues have also been linked with the development of these lesions [1]. Meyer first advocated the use of the term dermoid cyst for all developmental cysts on the floor of the mouth. They are classified into three histological types according to their contents: epidermoid cyst, dermoid cyst and teratoid cyst. Epidermoid cysts are lined by squamous epithelium and lack adnexal appendages, while dermoid cysts are lined by keratinizing squamous epithelium and contain adnexa-like sweat glands, sebaceous glands, and hair follicles. Teratoma consists of all three germinal layer derivatives [4,7].

Based on the relationship with the muscle of the floor of the mouth, Colp has provided an anatomic classification for these lesions into three types: those located below the mylohyoid muscle as ‘submental cysts’, those above the mylohyoid muscle as ‘sublingual cysts’ and the once located laterally beneath the tongue and above the mylohyoid in the submandibular space as ‘submandibular/lateral cyst. Our case was a sublingual epidermoid cyst [4,7].

Most of these tumours present as a painless asymptomatic swelling. In general, the patient becomes aware of the pathology due to the gradual deterioration of loco-regional functional signals such as difficulties related to speech, chewing, and swallowing. These symptoms are closely correlated to the size of the lesion. The development of the cyst is usually slow and does not involve painful symptoms, but it may become rapid and painful in the presence of concomitant infection. Epidermoid cysts are often tough to diagnose clinically because of their rare occurrence in this anatomic site and a close clinico-radiological resemblance with the more common differentials including ranulas and dermoid cysts. Fine needle aspiration cytology (FNAC) is often helpful in differentiating ranulas from dermoids based on the presence of mucoid content in ranulas versus keratinous material in dermoids [1,4].

Radiological evaluation is helpful in delineating the extension of the lesion and for surgical planning. Magnetic resonance imaging (MRI) and CT are more useful and allow for more accurate localization and extension of the tumour. MRI is frequently preferred over a CT scan as it is better in terms of soft tissue resolution and, thus, superiorly able to depict the internal content of the cyst. Nevertheless, none of the aspiration cytology, CT, or MRI techniques can differentiate with surety between the specific histological subtypes of dermoids. Thus, histopathological examination after excision of cysts is often required in equivocal cases [4,7,8].

In majority of the cases, enucleation is preferred if the cyst is large and symptomatic. However, giant extensive cysts abutting or involving vital structures might be considered for marsupialization. A different approach has been reported: intraoral, transcervical, and combined. The decision for surgical approaches is influenced by the size and site of tumour with the aim of complete removal and preventing recurrence. The intraoral approach is more suitable for sublingual epidermoid cysts and small/medium-sized lesions and offers the advantage of no external cosmetic scar. However, the intraoral approach offers limited exposure and is associated with higher morbidity linked with damage to Wharton’s duct and other vital structures in the sublingual space. Infra-mylohyoid cysts and large lesions are best approached by a transcervical incision for an optimal result [1,4,7,9]. An external approach was advocated, owing to the large cyst in our case. Malignant transformation into squamous cell carcinoma is seen in 5% of epidermoid cysts, although rare [10].

## Conclusions

An epidermoid cyst should be considered as a differential for any floor-of-mouth swelling. They are often clinico-radiologically confused with close differentials including ranulas and dermoid cysts. Aspiration cytology is a safe, cost-effective, and reliable tool in equivocal cases. Cyst excision is the most optimal treatment advocated and allows for microscopic evaluation. The histopathological examination allows for a confirmatory diagnosis and is possibly the only means of distinguishing between specific histological variants of dermoids.
